# Annual hepatitis C testing and positive tests among gay and bisexual men in Australia from 2016 to 2022: a serial cross-sectional analysis of sentinel surveillance data

**DOI:** 10.1136/sextrans-2024-056175

**Published:** 2024-06-20

**Authors:** Brendan L Harney, Rachel Sacks-Davis, Michael Traeger, Daniela K van Santen, Anna L Wilkinson, Jason Asselin, Christopher K Fairley, Norm Roth, Mark Bloch, Gail Matthews, Basil Donovan, Rebecca Guy, Margaret E Hellard, Joseph S Doyle

**Affiliations:** 1 Burnet Institute, Melbourne, Victoria, Australia; 2 Monash University, Melbourne, Victoria, Australia; 3 Department of Infectious Diseases, Alfred Health and Monash University, Melbourne, Victoria, Australia; 4 Harvard University, Cambridge, Massachusetts, USA; 5 Central Cllinical School, Monash University, Melbourne, Victoria, Australia; 6 Melbourne Sexual Health Centre, Alfred Health, Melbourne, Victoria, Australia; 7 Prahran Market Clinic, Melbourne, Victoria, Australia; 8 Holdsworth House Medical Clinic, Melbourne, Victoria, Australia; 9 The Kirby Institute, Kensington, New South Wales, Australia; 10 St Vincent's Hospital Sydney, Darlinghurst, New South Wales, Australia; 11 Sydney Sexual Health Centre, Sydney, New South Wales, Australia

**Keywords:** HEPATITIS C, HIV, Pre-Exposure Prophylaxis, Population Surveillance

## Abstract

**Objective:**

Guidelines recommend annual hepatitis C virus (HCV) testing for gay and bisexual men (GBM) with HIV and GBM prescribed HIV pre-exposure prophylaxis (PrEP). However, there is a limited understanding of HCV testing among GBM. We aimed to examine trends in HCV testing and positivity from 2016 to 2022.

**Methods:**

Using sentinel surveillance data, we examined the proportion of GBM with at least one test and the proportion with a positive test in each year for HCV antibody testing among GBM with no previous HCV positive test, HCV RNA testing among GBM with a positive antibody test but no previous positive RNA test (naïve RNA testing), and HCV RNA testing among people who had a previous RNA positive test and a subsequent negative test (RNA follow-up testing). Trends were examined using logistic regression from 2016 to 2019 and 2020 to 2022.

**Results:**

Among GBM with HIV, from 2016 to 2019 antibody testing was stable averaging 55% tested annually. Declines were observed for both naïve HCV RNA testing (75.4%–41.4%: p<0.001) and follow-up HCV RNA testing (70.1%–44.5%: p<0.001). Test positivity declined for HCV antibody tests (2.0%–1.3%: p=0.001), HCV RNA naïve tests (75.4%–41.4%: p<0.001) and HCV RNA follow-up tests (11.3%–3.3%: p=0.001). There were minimal or no significant trends from 2020 to 2022.

Among GBM prescribed PrEP, antibody testing declined from 2016 to 2019 (79.4%–69.4%: p<0.001) and was stable from 2020 to 2022. Naïve and follow-up HCV RNA testing was stable with an average of 55% and 60% tested each year, respectively. From 2016–2019, the proportion positive from HCV RNA naïve tests declined (44.1%–27.5%: p<0.046) with no significant change thereafter. Positive follow-up HCV RNA tests fluctuated with no or one new positive test among this group in most years.

**Conclusion:**

The proportion of GBM with positive HCV tests has declined, however a substantial proportion are not tested annually. A renewed focus on HCV testing, and treatment where required, is warranted to achieve HCV elimination among GBM in Australia.

**WHAT IS ALREADY KNOWN ON THIS TOPIC**

- Hepatitis C virus (HCV) microelimination, defined in this context as an 80% reduction in incidence by 2030 relative to 2015, is highly feasible among gay and bisexual men (GBM) in Australia, however achieving and sustaining this requires testing to provide treatment.
- There is a paucity of longitudinal studies focused on HCV testing data among GBM specific to the direct acting antiviral era in Australia, and globally.

**WHAT THIS STUDY ADDS**

- At a national and population level, this study shows that new positive HCV diagnoses have declined, but a substantial proportion of both GBM with HIV and GBM using pre-exposure prophylaxis are not tested annually and there have been declines in HCV testing.

**HOW THIS STUDY MIGHT AFFECT RESEARCH, PRACTICE OR POLICY**

- It is important to understand why a substantial proportion of GBM do not have annual HCV tests.
- Despite the observed declines in infection, low levels of ongoing testing, particularly follow-up RNA testing may have implications for the long-term sustainability of HCV microelimination among GBM in Australia.

## Background

Gay and bisexual men (GBM) with human immunodeficiency virus (HIV) are a key population at risk of hepatitis C virus (HCV) infection globally.^1^ However, with the advent of direct acting antiviral (DAA) HCV treatment, HCV microelimination, in this context defined as an 80% reduction in incidence by 2030 relative to 2015,^2^ appears feasible among GBM with HIV. This is supported by declines in HCV primary incidence among GBM with HIV internationally^3^ including Australia.^4^ Likewise, declines in reinfection incidence have also been reported in a number of countries,^5^ including Australia.^6^

Though historically GBM without HIV were considered at low risk of HCV infection, some evidence suggests GBM using HIV pre-exposure prophylaxis (PrEP) are also at increased risk of HCV,^1^ with both sexual behaviours and substance use behaviours associated with an increased risk of HCV infection.^7^ However, HCV prevalence and incidence among GBM prescribed PrEP is highly heterogenous, making it difficult to understand the level of HCV risk in this group.^8^

Guidelines in Australia and other high-income countries such as the USA and the UK recommend at least annual HCV testing for GBM with HIV and those using PrEP.^9–12^ Specific to Australia, HCV testing among GBM with HIV has long been recommended and early guidelines also recommended HCV testing for GBM prescribed PrEP.^13^ Although antibody testing is the first step in the HCV testing process, HCV RNA testing is required to identify active infection and is required following treatment or spontaneous clearance to detect possible reinfection among those at risk.^14^ Despite this, these guidelines to not explicitly provide guidance in relation to this.

There is a very limited understanding of HCV antibody and RNA testing, and resultant positive tests among GBM with HIV and GBM using HIV PrEP specific to the DAA era. Understanding this is important to identify potential challenges to achieving and sustaining HCV elimination among GBM in Australia. This study draws on data from a national sentinel surveillance system to examine the proportion of GBM with HIV and GBM prescribed PrEP who had HCV antibody and RNA tests and the proportion with positive tests across Australia from 2016 to 2022.

## Methods

### Data source

Data were from the Australian Collaboration for Coordinated Enhanced Sentinel Surveillance (ACCESS) of sexually transmissible infections (STIs) and bloodborne viruses (BBVs).^15^ ACCESS extracts data from electronic health records and links individual-level test results over time and across services including primary healthcare clinics, sexual health clinics and tertiary infectious disease outpatient clinics across Australia. Test results are extracted from electronic medical records as coded variables or electronic pathology reports. For the latter, natural language processing is applied to extract all results for relevant assays, and then an algorithm derived from the Communicable Disease Network of Australia case definition is applied to interpret results. Sites participating in ACCESS are selected based on providing clinical care to key population groups at risk of STIs and BBVs.

Among people with their sex recorded as male, GBM status was derived from being recorded as gay or bisexual in patient management systems or reporting one or more male partners in behavioural surveys at sexual health clinics. In addition, ever having had a rectal STI swab for chlamydia or gonorrhoea recorded was also used to categorise men as GBM, as previously validated.^16^ Dates of HIV diagnosis and electronic PrEP prescriptions were also extracted. Specific to these analyses, almost all sites are located in inner-suburban areas or large regional cities, reflecting the geographical distribution of GBM within Australia.

The requirement for individual-level consent was waived by all ethics committees, however patients may opt of out the system if requested.

### Outcomes

The outcomes were the proportion of GBM with at least one test, and of those the proportion with a new positive test in each calendar year from 2016 to 2022 for: (1) HCV antibody testing among GBM with no previous HCV positive test; (2) HCV RNA testing among GBM with a positive antibody test but no previous positive RNA test (naïve RNA testing); and (3) HCV RNA testing among people who had a previous RNA positive test and a subsequent negative test (follow-up RNA testing).

Data from 1 January 2012 onwards was used to determine HCV infection history, however analyses of testing and the proportion positive was from 2016 onwards as HCV DAA treatment first became widely available via Australia’s Pharmaceutical Benefit Scheme from 1 March 2016^14^ and PrEP became widely available in Australia via large implementation studies from mid-2016.^17 18^

### Inclusion criteria

GBM with HIV were included from the date of their first known HIV-positive test result and each year thereafter if they had at least one consultation recorded in ACCESS. We included GBM prescribed PrEP from their first recorded PrEP prescription date and each year thereafter if there was also a PrEP prescription recorded in ACCESS in that year as HCV testing is not recommended among HIV-negative GBM not using PrEP. Inclusion in each specific analysis was based on time-updated HCV antibody and/or HCV RNA test results. Though GBM could have had more than one HCV test per-year, each only contributed one data point per year to our analyses. (online supplemental figure 2).

10.1136/sextrans-2024-056175.supp1 Supplementary data

### Antibody testing

All GBM with no prior positive HCV antibody or RNA test result recorded in ACCESS were eligible for inclusion in analyses of antibody testing; this was inclusive of GBM who had no HCV test recorded. GBM who had a subsequent positive antibody test were removed from this component of the analysis after the first positive test as ongoing antibody testing among people with a previous positive antibody test *is not required* (online supplemental figure 2).

### Naïve HCV RNA testing

GBM with a positive antibody test were included if they had no HCV RNA positive test recorded in ACCESS. Those with a subsequent positive HCV RNA test were removed from this component of the analysis after the first positive test (online supplemental figure 1).

### Follow-up HCV RNA testing

GBM with a record of a positive HCV RNA test and a subsequent negative test were included in this analysis. Those with a subsequent HCV RNA positive test were removed from this component of the analysis after the positive test (online supplemental figure 3).

### Statistical analysis

Among GBM with at least one consultation in a calendar year, we estimated the proportion of GBM with an HCV test as applicable based on their HCV testing history. Of those with at least one test, we estimated the proportion of GBM with a new positive test; 95% CIs for proportions were calculated for each.

To examine trends in the proportion tested and the proportion positive, bivariate logistic regression without adjustment was used with year as a continuous independent variable where the OR represents the mean change in odds of the outcome (proportion tested or proportion positive) per year.^19^ Due to COVID-19, separate analyses were conducted to examine trends from 2016 to 2019 and 2020 to 2022.

All analyses were undertaken using Stata SE V.18.0 (College Station, Texas, USA).

## Results

### GBM with HIV

Between 1 January 2016 and 31 December 2022, 13 668 GBM with HIV attending 63 services had at least one consultation recorded, of whom 11 415 (83.5%) had at least one HCV antibody, qualitative HCV RNA or quantitative HCV RNA viral load test.

### HCV antibody testing

Among GBM with no previous record of a positive HCV antibody or HCV RNA test, the proportion of GBM tested averaged 54.7% between 2016 and 2019 with no significant change over time (OR 1.00, 95% CI 0.98 to 1.02) and 47.9% between 2020 and 2022 with a decline over time (OR 0.96, 95% CI 0.94 to 0.99) (table 1). Of those tested, the proportion with an HCV antibody positive test was highest in 2016 at 2% declining to 1.3% in 2019 (OR 0.83, 95% CI 0.74 to 0.92). The proportion positive declined to 0.7% in 2020 and was stable from 2020 to 2022 with no significant change over this time (OR 0.99, 95% CI 0.77 to 1.26 (table 1).

**Table 1:** HCV testing and new positive tests by calendar year among GBM with HIV in Australia 2016–2022

	Year	Consultation	Tested	% tested (95% CI)	Positive test	% positive (95% CI)
HCV antibody*	2016	8813	4747	53.9 (52.8 to 54.9)	95	2.0 (1.6 to 2.4)
	2017	9036	5042	55.8 (54.8 to 56.8)	63	1.2 (1.0 to 1.6)
	2018	9189	5005	54.5 (53.4 to 55.5)	44	0.9 (0.6 to 1.2)
	2019	9382	5109	54.5 (53.4 to 55.5)	65	1.3 (1.0 to 1.6)
	2020	9322	4441	47.6 (46.6 to 48.7)	33	0.7 (0.5 to 1.0)
	2021	8716	4367	50.1 (49.0 to 51.2)	33	0.8 (0.5 to 1.1)
	2022	8775	4025	45.9 (44.8 to 46.9)	29	0.7 (0.5 to 1.0)
Naïve HCV RNA†	2016	240	181	75.4 (69.5 to 80.7)	81	44.8 (37.4 to 52.3)
	2017	260	138	53.1 (46.8 to 59.3)	41	29.7 (22.2 to 38.1)
	2018	273	125	45.8 (39.8 to 51.9)	28	22.4 (15.4 to 30.7)
	2019	319	132	41.4 (35.9 to 47.0)	28	21.2 (14.6 to 29.2)
	2020	306	100	32.7 (27.5 to 38.2)	15	15.0 (8.6 to 23.5)
	2021	307	95	30.9 (25.8 to 36.4)	13	13.7 (7.5 to 22.3)
	2022	310	98	31.6 (26.5 to 37.1)	12	12.2 (6.5 to 20.4)
Follow-up HCV RNA‡	2016	405	284	70.1 (65.4 to 74.5)	32	11.3 (7.8 to 15.5)
	2017	547	333	60.9 (56.6 to 65.0)	14	4.2 (2.3 to 7.0)
	2018	592	285	48.1 (44.1 to 52.3)	16	5.6 (3.2 to 9.0)
	2019	609	271	44.5 (40.5 to 48.5)	9	3.3 (1.5 to 6.2)
	2020	606	210	34.7 (30.9 to 38.6)	4	1.9 (0.5 to 4.8)
	2021	608	234	38.5 (34.6 to 42.5)	1	0.4 (0.0 to 2.4)
	2022	610	194	31.8 (28.1 to 35.7)	7	3.6 (1.5 to 7.3)

*Among GBM with no previous positive HCV antibody or RNA test recorded in ACCESS (n=10 430).

†Among GBM with an antibody positive test and no previous positive HCV RNA test recorded in ACCESS (n=621).

‡Among GBM with a previous positive HCV RNA and at least one subsequent negative HCV RNA test recorded in ACCESS (n=798).

ACCESS, Australian Collaboration for Coordinated Enhanced Sentinel Surveillance; GBM, gay and bisexual men; HCV, hepatitis C virus.

### Naïve HCV RNA testing

Among GBM with a previous HCV antibody positive test but no record of a previous positive HCV RNA test, the proportion of GBM who had an HCV RNA test was highest in 2016 at 75.4% with a significant decline to 41.4% in 2019 (OR 0.64, 95% CI 0.58 to 0.72) (table 1). Testing declined further to 31.6% in 2022; however, the proportion tested was stable across 2020–2022 (OR 0.98, 95% CI 0.82 to 1.16). The proportion with an HCV RNA positive test declined from 44.8% in 2016 to 21.2% in 2019 (OR 0.67, 95% CI 0.57 to 0.79) and to 15% in 2020 remaining stable to 2022 at an average of 13.6% (OR 0.89, 95% CI 0.59 to 1.34) (table 1).

### Follow-up HCV RNA testing

Among GBM with a previous positive HCV RNA test and at least one subsequent negative test, follow-up HCV RNA testing declined from 70.1% in 2016 to 44.5% in 2019 (OR 0.69, 95% CI 0.64 to 0.75). Testing declined to 34.7% in 2020, with no significant change from 2020 to 2022 (OR 0.94, 95% CI 0.83 to 1.06). The proportion with a new positive RNA test was 11.3% in 2016 declining to 3.3% in 2019 (OR 0.69, 0.64, 0.75) and no significant trend from 2020 to 2022 (OR 0.94, 95% CI 0.83 to 1.06) (table 1).

### GBM prescribed HIV PrEP

#### Overall HCV testing

Between 1 January 2016 and 31 December 2022, 37 656 GBM attending 65 services were prescribed PrEP of whom 31 005 (82.3%) had at least one HCV antibody, qualitative HCV RNA or quantitative HCV RNA viral load test.

#### Antibody testing

Among those who had no record of a previous positive HCV test, the proportion who had an antibody test declined from 79.4% in 2016 to 69.4% in 2019 (OR 0.81, 95% CI 0.80 to 0.83) and was stable from 2020 to 2022 with an average of 66.1% tested each year (OR 0.99, 95% CI 0.96 to 1.01) (table 2). The proportion with a positive antibody test declined from 0.7% in 2016 to 0.3% in 2019 (OR 0.73, 95% CI 0.53 to 0.99) with no significant change thereafter (OR 0.52, 95% CI 0.24 to 1.11).

**Table 2:** HCV testing and new positive tests by calendar year among GBM prescribed PrEP in Australia (2016–2022)

	Year	Consultation	Tested	% tested (95% CI)	Positive test	% positive (95% CI)
HCV antibody*	2016	6780	5381	79.4 (78.4 to 80.3)	37	0.7 (0.5 to 0.9)
	2017	10 061	7957	79.1 (78.3 to 79.9)	33	0.4 (0.3 to 0.6)
	2018	13 446	9479	70.5 (69.7 to 71.3)	27	0.3 (0.2 to 0.4)
	2019	13 987	9705	69.4 (68.6 to 70.1)	28	0.3 (0.2 to 0.4)
	2020	13 422	8818	65.7 (64.9 to 66.5)	21	0.2 (0.1 to 0.4)
	2021	13 022	8772	67.4 (66.5 to 68.2)	12	0.1 (0.1 to 0.2)
	2022	14 294	9311	65.1 (64.4 to 65.9)	20	0.2 (0.1 to 0.3)
Naïve HCV RNA†	2016	55	34	61.8 (47.7 to 74.6)	15	44.1 (27.2 to 62.1)
	2017	67	42	62.7 (50.0 to 74.2)	19	45.2 (29.8 to 61.3)
	2018	68	39	57.4 (44.8 to 69.3)	10	25.6 (13.0 to 42.1)
	2019	66	40	60.6 (47.8 to 72.4)	11	27.5 (14.6 to 43.9)
	2020	72	38	52.8 (40.7 to 64.7)	9	23.7 (11.4 to 40.2)
	2021	61	29	47.5 (34.6 to 60.7)	4	13.8 (3.9 to 31.7)
	2022	62	26	41.9 (29.5 to 55.2)	2	7.7 (0.9 to 25.1)
Follow-up HCV RNA‡	2016	10	5	50.0 (18.7 to 81.3)	1	20.0 (0.5 to 71.6)
	2017	24	16	66.7 (44.7 to 84.4)	0	0.0 (0.0 to 20.6)
	2018	33	22	66.7 (48.2 to 82.0)	1	4.5 (0.1 to 22.8)
	2019	42	26	61.9 (45.6 to 76.4)	3	11.5 (2.4 to 30.2)
	2020	46	19	41.3 (27.0 to 56.8)	1	5.3 (0.1 to 26.0)
	2021	44	30	68.2 (52.4 to 81.4)	0	0.0 (0.0 to 11.6)
	2022	36	23	63.9 (46.2 to 79.2)	1	4.3 (0.1 to 21.9)

*Among GBM with no previous positive HCV antibody or RNA test recorded in ACCESS (n=33 848).

†Among GBM with an antibody positive test but no previous positive HCV RNA test recorded in ACCESS (n=255).

‡Among GBM with a previous positive HCV RNA and at least one subsequent negative HCV RNA test recorded in ACCESS (n=93).

ACCESS, Australian Collaboration for Coordinated Enhanced Sentinel Surveillance; GBM, gay and bisexual men; HCV, hepatitis C virus; PrEP, pre-exposure prophylaxis.

#### Naïve HCV RNA testing

Among those with an HCV antibody positive test but no record of a previous positive HCV RNA test, the proportion with an HCV RNA test was stable from 2016 to 2019 (OR 0.96, 95% CI 0.76 to 1.21) averaging 60.6% per year; 52.8% and 41.9% had a test in 2020 and 2022, respectively, however this decline was not statistically significant (OR 0.80, 95% CI 0.57 to 1.13) (table 2). The proportion with a positive HCV RNA test declined from 44% in 2016 to 27.5% in 2019 (OR 0.73, 95% CI 0.53 to 0.99); though there was a further decline from 23.7% in 2020 to 7.7% in 2022, this was not statistically significant (OR 0.52, 95% CI 0.24 to 1.11) (table 2).

#### Follow-up HCV RNA testing

Among GBM prescribed PrEP who had a history of a positive HCV RNA test and a subsequent negative test, an average of 60% had another RNA test per year with 2020 being a notable outlier at 41% (table 2). There was no significant trend from 2016 to 2019 (OR 1.05, 95% CI 0.71 to 1.56), however from 2020 there was a significant increase by 2022, rebounding to approximately 64% (OR 1.65, 95% CI 1.04 to 2.60) (table 2). There were none or only one person with a new positive HCV RNA test in most years, hence there was substantial fluctuation in the proportion with a new positive RNA test over time and no statistical analyses were undertaken (table 2).

## Discussion

Drawing on data from our national sentinel surveillance system with almost 13 700 GBM with HIV and 37 700 GBM prescribed HIV PrEP, we found that the proportion of HCV tests positive among GBM declined between 2016 and 2022, including for antibody, initial RNA testing and follow-up RNA testing. Though this is encouraging, a substantial proportion of GBM did not have an HCV test each year and there was some decline in testing.

Our study found that among GBM with HIV, an average of 52% were tested for HCV antibodies per year, which is higher than in previous research focused on HCV testing in this group. For example, in a US study, only 30% of GBM with HIV were tested for HCV antibody annually.^20^ In a Canadian study of HCV testing, inclusive of both antibody and RNA testing, testing peaked at 40% in 2015.^21^ We observed declines in HCV RNA testing over time. In people without a previous positive RNA test this fell from over 75% in 2016 to a nadir of 30.9% in 2021. While there was an encouraging decline in the number of people with a first HCV RNA positive diagnosis, around 13% of GBM with HIV tested HCV RNA positive in 2021 and 2022. While this is a low number overall, given the declines in testing, this may be a conservative estimate. In addition, RNA testing generally follows a positive antibody test. Given approximately 50% of GBM with HIV did not have an antibody test, there may be more with undiagnosed active HCV which cannot be ascertained from these data.

Among GBM with HIV who have had HCV treatment, there has been global concern about reinfection.^22^ Reinfection incidence in this group has been fairly low in Australia in the DAA era^23 24^ and there is some evidence of a decline in reinfection incidence.^6^ However, the detection of reinfection requires (the more expensive) RNA testing which declined among GBM with HIV in our study. While some of these RNA tests in earlier years may have been on treatment RNA testing and some people may no longer be engaging in behaviours that warrant testing, we are not able to ascertain this from these data.

Australian studies of PrEP included HCV testing and guidelines recommend at least annual HCV testing.^13 17 18^ As a result, HCV antibody testing was fairly high in 2016/2017 at approximately 80%. This aligns with self-report data from the UK where of 365 GBM using PrEP, 80.5% reported HCV testing in the prior 12 months.^25^ However, we observed declines in HCV antibody testing among GBM prescribed PrEP from 2016 to 2019. To the best of our knowledge, there are no other longitudinal studies specifically focused on HCV testing among GBM prescribed PrEP.

Among GBM prescribed PrEP who were eligible for inclusion based on their previous antibody and/or RNA test results, there was no significant decline in RNA testing. However, there were still a sizeable proportion of these men who were not tested for HCV RNA each year (although with small numbers available for analysis). Although a smaller group by overall numbers, the proportion with a new HCV RNA diagnosis in 2021, almost 14%, was similar to the 12% among GBM with HIV. Given many GBM prescribed PrEP did not have an RNA test, nor an antibody test to determine whether an RNA test is needed, it is also possible that these data are an underestimate of the total numbers with active HCV infection.

Arguably not all GBM actually need to be tested for HCV on an annual basis and further work is needed to understand the impact of focusing HCV testing on GBM at increased risk.^26^ Nonetheless, it is important to consider the reasons why a substantial proportion of GBM did not have HCV tests. The COVID-19 pandemic potentially had some impact from 2020 onwards, however particularly for RNA testing, there was indication of a decline in testing prior to 2020. Additionally, these analyses were specific to GBM who had at least one consultation and thus were engaged in care to at least some extent during this time. Undertesting seems unlikely to be due to a lack of awareness of HCV among clinicians: many clinics that contribute a substantial amount of these data were involved in HCV studies and PrEP implementation studies which included HCV testing. Another potential reason may be that HCV among GBM in Australia has largely been a ‘success story’ with significant declines in HCV prevalence and incidence, and relatively low incidence of reinfection among GBM with HIV.^4 23 24^ Likewise, prevalence and incidence among GBM prescribed PrEP has been very low,^27 28^ at least relative to some European settings.^7 29 30^ Accordingly, HCV may no longer be the focus for many clinicians. It is also possible that GBM and clinicians are making informed decisions about whether testing is needed, however there remains a limited understanding of to what extent GBM and clinicians discuss behaviours with specific regard to HCV and how this informs testing. In light of our findings, research exploring this is warranted.

Another potential reason is that unlike STI testing which is recommended up to every 3 months, HCV testing is only recommended annually.^9^ It is important to note however that STI testing has also been reported to have declined among GBM in Australia in recent years.^19^ Ascertaining whether HCV testing is due at any given clinic on an annual frequency may require additional review of clinical records. Specific to RNA testing, unless someone is currently being treated, HCV RNA testing in Australia is only reimbursed once per year. As a result, most laboratory services will not automatically conduct an RNA test on a positive antibody sample and a clinician needs to specifically request it. Further work is required to understand the cost-effectiveness and epidemiological impact of removing this restriction.

There are limitations to be considered in interpretation of our findings. First, we conducted this as a serial cross-sectional analysis and as such we did not account for the frequency of testing and how this may influence results as people testing more frequently may be different to those who test less frequently. Second, these data are limited to GBM attending this network of clinics which are highly concentrated in inner-suburban areas. As such, these data may not be representative of GBM attending clinics outside of this network in outer suburban or regional areas, or not attending clinics at all. Relatedly, GBM may have been tested for HCV at clinics that are not included in our sentinel surveillance network and there is the potential for unobserved tests. Similarly, GBM without HIV may have been prescribed PrEP at clinics that do not contribute data to this surveillance system or may have self-imported it and this would not be captured. Third, these data likely represent a ‘best case scenario’ as those included were GBM with at least one consultation and in the case of PrEP, at least one record of a prescription in the respective year. Finally, due to the limited availability and variation in behavioural and demographic data collected across services, we were not able to undertake analyses of HCV testing based on these. This limits identification of whether HCV testing is higher or lower among certain subgroups of GBM including those engaging in specific sexual and substance use behaviours.

## Conclusion

In our analysis of national sentinel surveillance data, there has been a decline in the proportion of GBM with new positive HCV tests, particularly HCV RNA tests. Testing has also declined overt ime and may be suboptimal to ensure early diagnosis and limit ongoing transmission. Though there is optimism that HCV microelimination is highly feasible among GBM in Australia, a renewed focus on appropriate HCV testing, and prompt treatment for anyone newly diagnosed, is likely warranted to ensure that this is achieved and sustained.

## Data Availability

Data are available upon reasonable request. Deidentified individual participant data included in this study cannot be shared publicly because of the sensitive nature of participant data anonymously extracted from participating clinical services. Access to deidentified data is available via the Burnet Institute, Melbourne, Victoria, Australia, with approval from the Alfred Hospital Human Research Ethics Committee for researchers who meet the criteria for access to confidential data. The ACCESS Study protocol has been published previously.
