# Supplementary material for: Annual hepatitis C testing and positive tests among gay and bisexual men in Australia from 2016 to 2022: a serial cross-sectional analysis of sentinel surveillance data
Source: Sex Transm Infect. 2024 Jun 20;100(5):295–301. doi: 10.1136/sextrans-2024-056175 (PMC11287637; doi:10.1136/sextrans-2024-056175)
Supplement: Supplementary data [file sextrans-2024-056175supp001.pdf]

ID	Event Date	HCV antibody	HCV RNA	PrEP prescription	HIV diagnosis date	Inclusion eligibility
8761349	05 Dec 2018	.	.	No	.	None
8761349	12 Mar 2019	Negative	.	Yes	.	Antibody testing
8761349	07 Aug 2019	.	.	Yes	.	Antibody testing
8761349	23 Dec 2019	.	.	Yes	.	Antibody testing
8761349	20 Jul 2020	.	.	No	.	None
8761349	22 Dec 2020	.	.	No	.	None
8761349	28 Nov 2021	Positive	.	Yes	.	Antibody testing
8761349	10 Dec 2021	.	Positive	Yes	.	Naive RNA testing
8761349	18 Mar 2022	.	Negative	Yes	.	None
8761349	13 Jul 2022	.	.	Yes	.	Follow-up RNA testing

Supplementary Figure 1. Hypothetical person whose first record is 5thth Dec 2018 with a no PrEP prescription is not included. They are eligible for the antibody testing analyses in 2019 as they have PrEP prescription data but not in 2020. Their progression through each testing definition is shown based on time updated HCV results when they have PrEP prescription data again in 2021 and 2022.

	Event Date	HCV antibody	HCV RNA	PrEP prescription	HIV diagnosis date	Inclusion eligibility
4356099	2 Mar 2016	.	.	No	.	None
4356099	21 Dec 2016	.	.	No	.	None
4356099	13 Jul 2017	Negative	.	No	13 Jul 2017	Antibody testing
4356099	9 Dec 2017	.	.	N/A	13 Jul 2017	Antibody testing
4356099	20 Jul 2018	.	.	N/A	13 Jul 2017	Antibody testing
4356099	22 Dec 2018	.	.	N/A	13 Jul 2017	Antibody testing
4356099	28 Jul 2019	Positive	.	N/A	13 Jul 2017	Antibody testing
4356099	14 Aug 2019	Positive	Negative	N/A	13 Jul 2017	Naive RNA testing
4356099	18 Dec 2019	.	.	N/A	13 Jul 2017	Naive RNA testing
4356099	3 Mar 2020	.	.	N/A	13 Jul 2017	Naive RNA testing
4356099	19 Jul 2020	.	.	N/A	13 Jul 2017	Naive RNA testing
4356099	20 Dec 2020	.	.	N/A	13 Jul 2017	Naive RNA testing
4356099	17 Jul 2021	Positive	Positive	N/A	13 Jul 2017	Naive RNA testing
4356099	30 Jul 2021	.	Positive	N/A	13 Jul 2017	None
4356099	28 Sep 2021	.	Negative	N/A	13 Jul 2017	None

Supplementary Figure 2. Hypothetical person whose first record is 2nd March 2016. They are not eligible for inclusion in any analysis until 13th July 2017 when they have a record of a HIV diagnosis. They are defined as having an HCV antibody test in 2017 and 2019 but *not* 2018. Following their first antibody positive test on 28^{thth} July 2019, they are no longer eligible for inclusion in the antibody testing analysis and become eligible for the initial HCV RNA testing analysis. They are defined as having an HCV RNA test in 2019 and 2021, but *not* in 2020. The are no longer eligible for the initial HCV RNA testing analysis following their first positive HCV RNA test result on 17th July 2021.

ID	Event Date	HCV antibody	HCV RNA	PrEP prescription	HIV diagnosis date	Inclusion eligibility
4560965	20 May 2016	Positive	Positive	N/A	10 Nov 2013	None
4560965	22 Dec 2016	.	Negative	N/A	10 Nov 2013	None
4560965	19 Jul 2017	.	.	N/A	10 Nov 2013	Follow-up RNA testing
4560965	23 Dec 2017	.	Negative	N/A	10 Nov 2013	Follow-up RNA testing
4560965	20 Jul 2018	.	.	N/A	10 Nov 2013	Follow-up RNA testing
4560965	22 Dec 2018	.	.	N/A	10 Nov 2013	Follow-up RNA testing
4560965	28 Jul 2019	.	.	N/A	10 Nov 2013	Follow-up RNA testing
4560965	10 Feb 2020	Positive	Positive	N/A	10 Nov 2013	Follow-up RNA testing
4560965	20 May 2020	.	Negative	N/A	10 Nov 2013	None

Supplementary Figure 3. Hypothetical person whose first record is 20th May 2016 with a positive HCV RNA test. They are eligible for the HCV RNA follow-up testing analysis *after* their first negative test on 22nd December 2016. They are defined as having an HCV RNA follow-up test in 2017 and 2020 but *not* in 2018 or 2019. Following their new RNA positive test on 10th February 2020, they are no longer eligible for inclusion in the follow-up HCV RNA testing analysis.
